# Maternal Tenofovir Disoproxil Fumarate Use in Pregnancy and Growth Outcomes among HIV-Exposed Uninfected Infants in Kenya

**DOI:** 10.1155/2015/276851

**Published:** 2015-12-28

**Authors:** Jillian Pintye, Agnes Langat, Benson Singa, John Kinuthia, Beryne Odeny, Abraham Katana, Lucy Nganga, Grace John-Stewart, Christine J. McGrath

**Affiliations:** ^1^Department of Global Health, University of Washington, Seattle, WA 98104, USA; ^2^Department of Nursing, University of Washington, Seattle, WA 98195, USA; ^3^United States Centers for Disease Control and Prevention (CDC), Nairobi 00202, Kenya; ^4^Center for Microbiology Research and Center for Clinical Research, Kenya Medical Research Institute, Nairobi 00202, Kenya; ^5^Department of Obstetrics & Gynecology, Kenyatta National Hospital, Nairobi 00202, Kenya; ^6^Department of Medicine, University of Washington, Seattle, WA 98195, USA; ^7^Department of Epidemiology, University of Washington, Seattle, WA 98195, USA; ^8^University of Texas Medical Branch, Galveston, TX 77555, USA

## Abstract

*Background*. Tenofovir disoproxil fumarate (TDF) is commonly used in antiretroviral treatment (ART) and preexposure prophylaxis regimens. We evaluated the relationship of prenatal TDF use and growth outcomes among Kenyan HIV-exposed uninfected (HEU) infants.* Materials and Methods.* We included PCR-confirmed HEU infants enrolled in a cross-sectional survey of mother-infant pairs conducted between July and December 2013 in Kenya. Maternal ART regimen during pregnancy was determined by self-report and clinic records. Six-week and 9-month* z*-scores for weight-for-age (WAZ), weight-for-length (WLZ), length-for-age (LAZ), and head circumference-for-age (HCAZ) were compared among HEU infants with and without TDF exposure using* t*-tests and multivariate linear regression models.* Results.* Among 277 mothers who received ART during pregnancy, 63% initiated ART before pregnancy, of which 89 (32%) used TDF. No differences in birth weight (3.0 kg versus 3.1 kg, *p* = 0.21) or gestational age (38 weeks versus 38 weeks, *p* = 0.16) were detected between TDF-exposed and TDF-unexposed infants. At 6 weeks, unadjusted mean WAZ was lower among TDF-exposed infants (−0.8 versus −0.4, *p* = 0.03), with a trend towards association in adjusted analyses (*p* = 0.06). There were no associations between prenatal TDF use and WLZ, LAZ, and HCAZ in 6-week or 9-month infant cohorts.* Conclusion.* Maternal TDF use did not adversely affect infant growth compared to other regimens.

## 1. Introduction

Tenofovir disoproxil fumarate- (TDF-) containing combination antiretroviral therapy (ART) is currently considered a first-line regimen for HIV treatment and prevention of mother-to-child transmission (PMTCT) Option B/B+ by the World Health Organization (WHO) [1]. TDF and the fixed-dose combination of emtricitabine (FTC) 200 mg and TDF 300 mg are also recommended by WHO for antiretroviral preexposure prophylaxis (PrEP) in key populations, including women in HIV-serodiscordant couples who may wish to conceive [2]. TDF is considered a Pregnancy Category B drug, which means that no adequate evidence of risk in humans has been established by the United States Food and Drug Administration (FDA) [3]. The FDA recommends TDF as an alternative nucleotide analogue reverse transcriptase inhibitor (NRTI) for HIV-infected antiretroviral-naïve pregnant women due to limited data on TDF safety during pregnancy [4]. Animal studies in macaques have found adverse effects of high-dose TDF during pregnancy on bone mineralization and intrauterine growth measured at birth, but these effects were not observed at lower doses, which are more consistent with human TDF use [5–8]. As ART and PMTCT Option B/B+ programs expand and PrEP accessibility scales up, the likelihood of pregnant women using TDF will increase and obtaining safety information on TDF use during pregnancy will have important public health implications [9].

Several studies [10–20] and one systematic review [9] reported that prenatal TDF use for HIV treatment generally appears to be safe for pregnancy outcomes. Additionally, the most recent report from the Antiretroviral Pregnancy Registry showed no evidence of increased birth defects among 1,982 infants born to HIV-infected women in the United States who took TDF during their first trimester [21]. Limited data are available on the safety of TDF use for PrEP in pregnancy, though small studies suggest no difference in birth outcomes between mothers with and without short-term prenatal PrEP use [22, 23]. However, few studies have assessed the effects of prolonged prenatal TDF use on postnatal infant growth and bone health, and these have mixed results [14, 18, 19, 24, 25]. Only one small study evaluated prolonged prenatal TDF use and infant growth outcomes in a sub-Saharan African cohort indicating a need for additional data from this setting [26]. Data from HIV-exposed uninfected (HEU) infants could be particularly useful when assessing safety of prenatal TDF use for PrEP. We aimed to evaluate the relationship of prenatal TDF use and growth outcomes among HEU infants born to mothers who used combination ART for PMTCT or HIV treatment during pregnancy in Kenya.

## 2. Materials and Methods

### 2.1. Study Design and Participants

Data, from participants enrolled in two cross-sectional surveys evaluating the national PMTCT program and maternal-child health (MCH) indicators in Kenya conducted between June and December 2013, were analyzed for this study. The first survey used probability proportionate to size sampling to select 121 MCH clinics in seven of the eight geographical regions in Kenya from which all mother-infant pairs were sampled to participate during a 5-day period per clinic. The second survey sampled only HIV-infected women attending 30 MCH clinics in Nyanza province during a fixed 10-day period. In total, 140 clinics were sampled as some clinics were selected for both surveys. Women were eligible to be included in the survey if they were willing and able to provide informed consent and their infant was attending clinic to receive week 6 or month 9 immunizations. Infants were excluded if they were brought to the clinic by someone other than their biological mother or informed consent from their mother was not provided.

At enrollment into both surveys, a nurse administered the study questionnaire and obtained anthropometric measurements of the mother and infant. Mothers were identified as HIV-infected through self-report. HIV status during pregnancy and timing of HIV diagnosis were confirmed using MCH booklets, a form of clinical records used in Kenya which documents MCH and HIV services received in pre-/postnatal care. All mothers identified as HIV-infected were offered infant HIV testing and a dried blood spot (DBS) sample was taken for HIV DNA PCR testing after consent. All infants with PCR-confirmed HIV-negative serostatus and complete anthropometric measurements born to HIV-positive mothers with documented use of 3-drug combination ART during pregnancy were included in this analysis.

### 2.2. Data Collection

Study questionnaires obtained information on maternal sociodemographics, sexual behaviors, and medical history. Data on infant birth and medical characteristics was also collected. Maternal body mass index (kilograms/meters^2^) was calculated from height and weight measurements ascertained by study nurses at questionnaire administration. MCH booklets confirmed clinical data self-reported on questionnaires. Data were abstracted from MCH booklets if mothers were not sure of ART regimen used during pregnancy, WHO clinical stage, last CD4 count, infant birth weight, or gestational age at birth. Trimester of ART initiation was calculated using the date of ART initiation and infant birth date, as documented in MCH booklets. All mothers with confirmed ART initiation prior to pregnancy were considered to have first trimester ART use. Maternal prenatal TDF use was defined as a documented use of TDF-containing ART regimen for any amount of time during pregnancy.

### 2.3. Outcome Measures

Trained study nurses obtained standardized anthropometric measurements from each infant, including length in centimeters (cm), weight in kilograms (kg), and head circumference in cm [27].* z*-scores for weight-for-age (WAZ), weight-for-length (WLZ), length-for-age (LAZ), and head circumference-for-age (HCAZ) were calculated using the WHO Child Growth Standards in WHO Anthro software [27, 28].

### 2.4. Statistical Methods

All HIV-infected mother-HEU infant pairs with information on prenatal 3-drug combination ART regimen type and anthropometric measurements were included in this analysis; HIV-infected mothers without information on ART regimen type and verification from their MCH booklets were excluded. Separate analyses were conducted for infants attending 6-week and 9-month immunization visits. Chi-squared tests for proportions and Kruskal-Wallis tests for continuous measures were used to detect differences in sociodemographic and medical characteristics among mother-infant pairs with and without prenatal TDF use. Growth outcomes among HEU infants with and without maternal prenatal TDF use were compared using* t*-tests and multivariate linear regression for continuous measures of weight (kg), length (cm), head circumference (cm), WAZ, WLZ, LAZ, and HCZ. Characteristics associated with growth faltering, defined as WAZ, WLZ, LAZ, and HCZ < −2 standard deviations (SD), were assessed using Chi-squared tests and multivariate logistic regression. All linear and logistic regression models accounted for clustering at the clinic level.

We determined* a priori* to adjust our statistical models for maternal age, education level, BMI, time since HIV diagnosis, and infant breastfeeding and gestational age at birth due to the known associations of these factors with TDF use or infant growth outcomes [29–32]. Additionally, we identified several demographic, behavioral, and medical characteristics to assess as potential confounders: WHO clinical stage, number of living children, marital status, marriage type (monogamous versus polygamous), enrollment site in Nyanza (a culturally distinct region with high HIV prevalence), ever having received CD4 testing, last CD4 count (cell/*μ*L) during pregnancy, and trimester of first combination ART regimen use during pregnancy and protease inhibitor- (PI-) containing ART regimen (versus no PI). Additional potential confounders were included in the final models if they substantially changed the logistic regression model odds ratio or linear regression coefficient (>10% change). Multivariate risk scores were used to simultaneously adjust for maternal age, maternal education level, breastfeeding, gestational age at birth, time since maternal HIV diagnosis, maternal WHO clinical stage, timing of ART initiation (before or during pregnancy), and trimester of first use of 3-drug combination ART regimen during pregnancy and PI-containing ART regimen in final models. Multivariate risk scores were used to impute missing data for adjustment in multivariate models. The validity and details regarding this approach have been described elsewhere [33, 34]. These scores were included in the final models as quintiles.

To examine our statistical models with the most precision for first trimester 3-drug combination ART exposure, we restricted our dataset to only mother-infant pairs with documented ART initiation prior to pregnancy. Current WHO Child Growth Standards calculate age and sex-adjusted* z*-scores based on infants born >37 weeks of gestation and therefore potential nondifferential* z*-score misclassification may occur in preterm infants (gestational age <37 weeks) [35]. To examine the robustness of our multivariate regression models without the effect of prematurity, we repeated the primary analysis restricted to infants born >37 weeks of gestation. We also repeated the primary analysis using indicator variables for missing values to account for the potential categorical effect of missing data. Data were analyzed using STATA 13.1/MP for Windows (Stata Corporation, College Station, TX).

### 2.5. Ethical Considerations

The study was approved by the institutional review boards of the 3 collaborating institutions including the Kenya Medical Research Institute, the University of Washington, and the US Centers for Disease Control and Prevention.

## 3. Results

### 3.1. Enrollment Characteristics

A total of 277 HIV-infected mothers and their HEU infants (56% of all HEU infants in both surveys) had documented 3-drug combination ART use during pregnancy that met criteria for inclusion in this analysis; 155 (56%) attend 6-week infant immunizations; 122 (44%) attend 9-month infant immunizations. Most mothers were married (84%), the median age was 29 years (interquartile range (IQR) 25–34), and the median time since HIV diagnosis was 8 years (IQR 5–8). Over half of the mothers (64%) initiated 3-drug combination ART before pregnancy and 89 (32%) used a TDF-containing regimen at any time during pregnancy. Among mothers that did not use TDF-containing regimens, the most common combination ART was zidovudine, lamivudine, and nevirapine (AZT/3TC/NVP) (78%) followed by stavudine, lamivudine, and nevirapine (d24/3TC/NVP) (8%). Tenofovir, lamivudine, and nevirapine (TDF/3TC/NVP) and tenofovir, lamivudine, and efavirenz (TDF/3TC/EFV) were the most common regimens among mothers who used TDF-containing ART (65% and 26%, resp.). Mothers with and without prenatal TDF use had similar sociodemographic characteristics (Table 1). Compared to mothers without prenatal TDF use (*n* = 188), mothers with prenatal TDF use (*n* = 89) were more likely to receive PIs (26% versus 7%, *p* < 0.001), were more likely to be WHO clinical stage III (14% versus 6%, *p* = 0.030), and had modestly lower median BMI (22 versus 23, *p* = 0.031). There was no difference in median time since ART initiation between mothers with and without TDF use that initiated 3-drug combination ART prior to pregnancy (42 versus 36 months, *p* = 0.654). Similarly, mothers with and without TDF use that initiated 3-drug combination ART in pregnancy (*n* = 76) did not have a significant difference in median time since ART initiation (6 versus 9 months, *p* = 0.809).

Most infants were currently breastfeeding (87%) and half (51%) were male. Mean gestational age at birth was similar for infants with and without mothers that used TDF during pregnancy (37.8 weeks versus 38.1, *p* = 0.337). We did not detect differences in mean birth weight (3.0 kg versus 3.2 kg, *p* = 0.14) or prevalence of low birth weight <2.5 kg (10% versus 7%, *p* = 0.449) among infants with and without prenatal TDF exposure.

### 3.2. Growth Outcomes among HEU Infants Attending 6-Week Visits

We detected a modest difference in mean weight (4.3 kg versus 4.7 kg, *p* = 0.015, Table 2) and WAZ (−0.8 versus −0.4, *p* = 0.033) between infants attending 6-week visits with* in utero* TDF exposure compared to infants without exposure to TDF. There was no detectable difference between prenatal TDF use and WAZ < −2 SD among infants attending 6-week visits (12% versus 7%, *p* = 0.288). There were no detectable differences for WLZ (0.3 versus 0.6, *p* = 0.462), WLZ < −2 SD (10% versus 16%, *p* = 0.317), length (52.8 cm versus 53.0 cm, *p* = 0.766), LAZ (−1.2 versus −1.2, *p* = 0.951), and LAZ < −2 SD (37% versus 38%, *p* = 0.976). There were also no differences in head circumference among HEU infants attending 6-week visits with and without prenatal TDF exposure.

After adjustment for maternal age, maternal education, breastfeeding, gestational age at birth, time since maternal HIV diagnosis, maternal WHO clinical stage, timing of ART initiation (before or during pregnancy), and trimester of first combination ART regimen use during pregnancy and PI-containing ART, we found no association between maternal prenatal TDF use and weight, length, and HC among infants attending 6-week visits (Table 3): WAZ (adjusted coefficient (adj. coeff.) = −0.46, 95% confidence interval (CI): −0.93, 0.01, *p* = 0.057); WLZ (adj. coeff. = −0.30, 95% CI: −1.16, 0.56, *p* = 0.483); LAZ (adj. coeff. = −0.0, 95% CI: −0.83, 0.83, *p* = 0.992); HCZ (adj. coeff. = −0.02, 95% CI: −0.76, 0.71, *p* = 0.948). We also found no association between* z*-scores <−2 SD and maternal prenatal TDF use for WAZ (adjusted odds ratio (aOR) = 1.9, 95% CI: 0.5, 6.4, *p* = 0.321), WLZ (aOR = 0.6, 95% CI: 0.2, 1.9, *p* = 0.374), LAZ (aOR = 1.0, 95% CI: 0.5, 2.1, *p* = 0.941), or HCZ (aOR = 2.1, 95% CI: 0.3, 16.1, *p* = 0.483). Because maternal BMI and maternal WHO stage were collinear, a separate multivariate model was constructed including all covariates except for WHO stage. The association between maternal prenatal TDF use and WAZ remained non-significant (adj. coeff. = −0.4, 95% CI: −0.9, 0.2, *p* = 0.192).

### 3.3. Growth Outcomes among HEU Infants Attending 9-Month Visit

Among infants receiving 9-month immunizations, we did not detect differences for any measure of weight between HEU infants with or without mothers that used TDF during pregnancy (Table 3): weight (8.1 kg versus 8.4 kg, *p* = 0.302); WAZ (−0.6 versus −0.3, *p* = 0.306); WAZ < −2 SD (18% versus 12%, *p* = 0.336); WLZ (0.1 versus 0.4, *p* = 0.597); and WLZ < −2 SD (13% versus 9%, *p* = 0.431). Similarly, we did not detect differences between length or head circumference.

Among infants attending 9-month visits, we found no association between weight, length, or HC growth indicators and whether or not mothers had used TDF during pregnancy after adjustment (Table 3): WAZ (adjusted coefficient [adj. coeff.] = −0.31, 95% CI: −0.97, 0.35, *p* = 0.349); WLZ (adj. coeff. = −0.22, 95% CI: −1.19, 0.76, *p* = 0.655); LAZ (adj. coeff. = −0.35, 95% CI: −1.40, 0.71, *p* = 0.514); HC (adj. coeff. = −0.07, 95% CI: −1.04, 0.90, *p* = 0.888). Similarly, we did not find any association between* z*-scores <−2 SD and maternal prenatal TDF use for WAZ (aOR = 1.6, 95% CI: 0.6, 4.6, *p* = 0.378), WLZ (aOR = 1.6, 95% CI: 0.5, 5.9, *p* = 0.452), LAZ (aOR = 1.9, 95% CI: 0.8, 4.5, *p* = 0.147), or HCZ (aOR = 1.3, 95% CI: 0.3, 5.3, *p* = 0.684). When substituting maternal BMI for maternal WHO stage, the association between maternal prenatal TDF use and WAZ remained non-significant (adj. coeff. = −0.4, 95% CI: −1.2, 0.4, *p* = 0.319).

### 3.4. Sensitivity Analyses

When restricting our dataset to only mother-infant pairs with documented 3-drug combination ART initiation prior to pregnancy (*n* = 176), we found that maternal prenatal TDF use was associated with a trend for lower absolute WAZ (crude coeff. = −0.59, 95% CI: −1.17, 0.02, *p* = 0.044), similar to our primary results. In adjusted models, we did not detect significant associations between maternal prenatal TDF use and any growth indicator, though our power to detect associations was reduced. To reduce the effect of potentially misclassified* z*-scores for preterm infants, we repeated the primary analysis excluding HEU infants from the overall study population born ≤37 weeks of gestation (*n* = 63). Among HEU infants attending 6-week visits born >37 weeks of gestation (*n* = 91), we detected a modest difference in mean weight (4.4 kg versus 4.8 kg, *p* = 0.011) and mean WAZ (−0.3 versus −0.8, *p* = 0.021) between those born to mothers with and without prenatal TDF use. After adjustment, maternal prenatal TDF use was not associated with WAZ (adj. coeff. = −0.5, 95% CI: −0.9, 0.04, *p* = 0.072) or WAZ < −2 SD (aOR = 2.0, 95% CI: 0.4, 10.2, *p* = 0.418) among HEU infants born >37 weeks of gestation. Results for length, HC, WLZ, LAZ, and HCZ excluding infants born <37 weeks of gestation did not have appreciable differences with those of the full study population for infants attending 6-week or 9-month visits (data not shown). Results using indicator variables for missing values to account for the potential categorical effect of missing data were similar to our primary results (data not shown).

## 4. Discussion

Given the increasing use of TDF for HIV treatment and biomedical prevention strategies in sub-Saharan Africa, further evaluation on postnatal effects of prenatal TDF use in this setting is crucial. While data on PrEP use in pregnancy accumulates, current data available from HEU infants born to mothers on TDF-containing ART regimens may potentially contribute to the growing safety profile of prolonged maternal prenatal TDF use on infant growth outcomes. In this study of HEU infants in Kenya, we found marginal differences in weight and WAZ between infants attending 6-week visits born to mothers with and without TDF use during pregnancy. After adjustment for sociodemographic and medical characteristics, prenatal TDF use was associated with a trend for modest decrease in weight or WAZ. We found no association of prenatal TDF use with length, WLZ, LAZ, HC, or HCZ among infants attending 6-week or 9-month visits. Our data contribute to the limited number of studies investigating safety of TDF on postnatal growth outcomes among sub-Saharan African HEU populations [26].

Growth indicators, specifically height and HAZ, may provide important information on prenatal TDF use and infant bone health in settings where bone mineralization tests are not readily available. Similar to Gibb et al. (2012), which examined a population (*n* = 182) of PCR-confirmed negative HEU infants in Uganda and Zimbabwe, we did not find differences in height or HAZ among infants with and without maternal TDF during pregnancy [26]. A larger cohort study (*n* = 2029) in the United States detected slightly lower infant length at 12 months of age between infants with and without* in utero* TDF exposure (LAZ −0.17 versus −0.03, *p* = 0.04). The long-term clinical relevance of this modest difference is not well understood.

WHO Child Growth Standards, commonly used in clinical settings of Kenya and other sub-Saharan African countries, relate observed growth parameters (height, weight, HC, and middle upper arm circumference) to those expected in normal children according to percentiles using* z*-scores [27]. However, current WHO Child Growth Standards are calibrated for infants born >37 weeks and do not account for growth trajectories of preterm infants which differ from term infants [28, 35]. This may lead to misclassification of growth faltering among infants born ≤37 weeks. Other studies investigating safety of TDF use during pregnancy have used alternative growth charts that account for gestational age at birth [24, 25]. However, these methods have not been validated in sub-Saharan Africa where WHO Child Growth Standards are typically used. To our knowledge, this is the first study evaluating prenatal TDF use and growth outcomes among HEU in Africa to incorporate the potential effect of prematurity on postnatal growth outcomes. A systematic review and meta-analysis reported that 12% of infants in sub-Saharan Africa are born preterm [36]. In our study, which included only infants born to HIV-infected mothers, ~24% of infants were born ≤37 weeks of gestation, similar to other studies of HIV-exposed infants in sub-Saharan Africa [37, 38]. Future evaluations of prenatal TDF use on infant growth outcomes in this setting should make analytic considerations for* z*-scores of preterm infants when accurate gestational age at birth information is available. Forthcoming international growth standards for weight, length, and head circumference by gestational age and sex developed by the INTERGROWTH-21st Project may be particularly useful for evaluating postnatal growth in settings with high prevalence of birth ≤37 weeks of gestation in Africa [39].

Our study has limitations that should be noted. The relatively small sample size may have limited our power to detect statistical differences and associations, though most studies examining prenatal TDF use and growth outcomes have included fewer infants [14, 18, 19, 26]. As roll out of TDF as a first-line PMTCT Option B/B+ scales up and longitudinal data becomes available, larger prospective studies will remain important in evaluating the safety of prenatal TDF use. TDF exposure was determined by self-report and clinical records in our study. This limited our ability to precisely investigate the association between timing of* in utero* TDF exposure, fetal development, and subsequent growth outcomes. Data from future prospective studies that follow ART or TDF naïve women that initiate TDF use during pregnancy will be especially valuable as timing of TDF exposure as it relates to fetal bone development is not well understood.

## 5. Conclusions

Our findings add to previous studies, indicating that prenatal TDF use appears to be safe compared to non-TDF-containing ART regimens. More specifically, our study contributes to the very limited data available on safety of TDF use and growth outcomes in Africa where TDF-containing regimens are expanding for HIV treatment and PMTCT. PrEP for HIV-uninfected women during pregnancy may have additional benefit in Africa where maternal seroconversion during pregnancy and breastfeeding contributes significantly to the pediatric HIV burden [40]. Further research on long-term effects of maternal prenatal TDF use, particularly from mothers using PrEP in pregnancy, is vital as TDF use rapidly scales up.

## Figures and Tables

**Table 1:** Distribution of demographic and medical characteristics by any maternal prenatal TDF use, among HEU infants exposed for combination ART^1^.

	Median (IQR) or *N* (percentage)					
	Mother-infant pairs at 6-week visit (*n* = 155)			Mother-infant pairs at 9-month visit (*n* = 122)		
	Maternal TDF use during pregnancy^2^			Maternal TDF use during pregnancy^2^		
	Yes (*n* = 51)	No (*n* = 104)	*p* value^3^	Yes (*n* = 38)	No (*n* = 84)	*p* value^3^
Maternal demographic characteristics						
Age (years)	28 (24–33)	28 (25–33)	0.730	30 (26–35)	31 (25–34)	0.930
Education completed (years)	8 (7–11)	8 (7–10)	0.430	8 (8–11)	8 (7–12)	0.901
Number of children	3 (2–4)	3 (2–4)	0.109	4 (2–4)	3 (2–4)	0.460
Married/cohabiting	45 (88%)	85 (82%)	0.301	32 (84%)	71 (85%)	0.965
Monogamous marriage (versus polygamous)	36 (86%)	67 (80%)	0.415	25 (81%)	56 (80%)	0.940
Enrollment site in Nyanza (versus outside Nyanza)	40 (78%)	67 (64%)	0.076	28 (74%)	63 (75%)	0.877

Maternal medical characteristics						
Time since first HIV diagnosis (years)	8 (5–8)	8 (4–8)	0.862	8 (6–8)	8 (7-8)	0.838
Initiated ART before pregnancy (versus during pregnancy)	30 (60%)	69 (75%)	0.063	30 (79%)	47 (65%)	0.137
Ever received CD4 testing	45 (94%)	97 (97%)	0.348	38 (100%)	81 (96%)	0.238
Last CD4 (cell/*µ*L) during pregnancy	365 (268–520)	395 (273–553)	0.589	481 (326–600)	550 (368–741)	0.144
Maternal WHO clinical stage						
Stage 1	16 (32%)	25 (24%)	0.311	14 (37%)	29 (35%)	0.804
Stage 2	8 (16%)	20 (19%)	0.608	8 (21%)	15 (18%)	0.676
Stage 3	8 (16%)	10 (10%)	0.257	4 (11%)	1 (1%)	**0.016** ^*∗*^
Unknown	18 (36%)	48 (47%)	0.214	12 (32%)	39 (46%)	0.124
PI-containing maternal ART regimen	16 (31%)	7 (7%)	**<0.001** ^*∗*^	7 (18%)	7 (8%)	0.105
Trimester of first combo ART use						
1st trimester^4^	39 (89%)	82 (94%)	0.253	33 (97%)	58 (94%)	0.459
2nd trimester	3 (7%)	2 (2%)	0202	0 (0%)	1 (2%)	0.457
3rd trimester	2 (5%)	3 (3%)	0.416	1 (3%)	3 (5%)	0.682
Body mass index (kg/m^2^)	23 (20–25)	23 (21–25)	0.193	21 (20–24)	23 (20–25)	**0.005** ^*∗*^

Infant characteristics						
Gestational age at birth (weeks)	38 (36–39)	38 (36–40)	0.562	38 (37–39)	38 (37–40)	0.121
Birth weight (kilograms)	3.0 (2.7–3.5)	3.1 (2.8–3.5)	0.338	3.3 (2.5–3.5)	3.1 (2.8–3.7)	0.363
Infant male sex	35 (49%)	59 (57%)	0.365	23 (61%)	35 (42%)	0.053
Currently breastfeeding	49 (98%)	99 (99%)	0.615	31 (84%)	57 (68%)	0.070

^*∗*^ *p* < 0.05.

^1^Missing data not shown.

^2^Maternal TDF use during pregnancy defined as any reported TDF-containing regimen used at any time during pregnancy among mothers that used combination ART.

^3^Chi-squared test for proportions or Kruskal-Wallis test for continuous measures.

^4^Including women that initiated ART before pregnancy.

**Table 2:** Distribution of mean weight, length, and head circumference (HC) anthropometric measurements and age and sex-adjusted *z*-scores among HEU infants attending 6-week and 9-month immunization visits, by maternal TDF use in pregnancy.

Anthropometric measure	Infants attending 6-week visit				Infants attending 9-month visit			
	Mean (95% CI) or *N* (%)				Mean (95% CI) or *N* (%)			
	Total (*n* = 155)	No maternal TDF use during pregnancy (*n* = 104)	Any maternal TDF use during pregnancy^1^ (*n* = 51)	*p* value^2^	Total (*n* = 122)	No maternal TDF use during pregnancy (*n* = 84)	Any maternal TDF use during pregnancy^1^ (*n* = 38)	*p* value^2^
Weight								
Absolute weight-(kg)	4.6 (4.4, 4.7)	4.7 (4.5, 4.8)	4.3 (4.1, 4.6)	**0.015** ^*∗*^	8.3 (8.1, 8.5)	8.4 (8.1, 8.7)	8.1 (7.7, 8.6)	0.302
Absolute WAZ	−0.5 (−0.7, −0.3)	−0.4 (−0.6, −0.1)	−0.8 (−1.2, −0.5)	**0.033** ^*∗*^	−0.4 (−0.6, −0.1)	−0.3 (−0.6, 0.0)	−0.6 (−1.2, 0.0)	0.306
WAZ < −2 SD	13 (8%)	7 (7%)	6 (12%)	0.288	17 (14%)	10 (12%)	7 (18%)	0.336
Absolute WLZ	0.5 (0.1, 0.9)	0.6 (0.1, 1.1)	0.3 (−0.3, 0.9)	0.462	0.3 (−0.1, 0.7)	0.4 (−0.2, 0.9)	0.1 (−0.5, 0.8)	0.597
WLZ < −2 SD	21 (14%)	16 (16%)	5 (10%)	0.317	12 (10%)	7 (9%)	5 (13%)	0.432

Length								
Absolute length (cm)	52.9 (51.2, 53.7)	53.0 (52.0, 54.0)	52.8 (51.6, 53.9)	0.766	68.1 (66.9, 69.2)	68.2 (66.7, 69.7)	67.7 (66.3, 69.2)	0.710
Absolute LAZ	−1.2 (−1.5, −0.8)	−1.2 (−1.6, −0.7)	−1.2 (−1.7, −0.6)	0.951	−1.0 (−1.5, −0.5)	−1.0 (−1.7, −0.3)	−1.1 (−1.9, −0.3)	0.797
LAZ < −2 SD	58 (37%)	39 (38%)	19 (37%)	0.976	36 (30%)	22 (26%)	14 (37%)	0.232

Head circumference (HC)								
Absolute HC (cm)	38.7 (38.3, 39.0)	38.7 (38.3, 39.1)	38.5 (37.9, 39.1)	0.596	44.0 (43.6, 44.4)	44.0 (43.5, 44.5)	44.0 (43.2, 44.8)	0.995
Absolute HCZ	0.7 (0.4, 1.0)	0.7 (0.4, 1.1)	0.7 (0.2, 1.2)	0.892	−0.3 (−0.7, 0.0)	−0.3 (−0.7, 0.1)	−0.4 (−1.1, 0.4)	0.873
HCZ < −2 SD	4 (3%)	2 (2%)	2 (4%)	0.461	22 (18%)	14 (17%)	8 (21%)	0.560

SD = standard deviation.

^*∗*^ *p* < 0.05.

^1^Maternal TDF use defined as any reported TDF-containing ART regimen used for any amount of time during pregnancy among mothers that used combination ART while pregnant.

^2^Chi-squared test for proportions or 2-sample *t*-test for means.

**Table 3:** Association of age and sex-adjusted *z*-scores for weight, weight-for-length, length, and head circumference (HC) among HEU infants and maternal TDF use in pregnancy, by 6-week and 9-month immunization visits^1^.

Growth outcome	Infants attending 6-week immunization visit				Infants attending 9-month immunization visit			
	Univariate^2^		Multivariate^2,3^		Univariate^2^		Multivariate^2,3^	
	Coeff. or OR (crude) (95% CI)	*p* value	Coeff. or OR (adj) (95% CI)	*p* value	Coeff. or OR (crude) (95% CI)	*p* value	Coeff. or OR (adj.) (95% CI)	*p* value
Weight								
Absolute WAZ	−0.46 (−0.93, 0.01)	0.056	−0.46 (−0.93, 0.01)	0.057	−0.31 (0.96, 0.34)	0.341	−0.31 (−0.97, 0.35)	0.349
WAZ < −2 SD	1.84 (0.55, 6.23)	0.322	1.86 (0.54, 6.35)	0.321	1.67 (0.59, 4.72)	0.333	1.60 (0.56, 4.56)	0.378
Absolute WLZ	−0.31 (−1.15, 0.53)	0.461	−0.30 (−1.16, 0.56)	0.483	−0.24 (−1.19, 0.71)	0.608	−0.22 (−1.19, 0.76)	0.655
WLZ < −2 SD	0.58 (0.18, 1.93)	0.377	0.59 (0.17, 1.93)	0.374	1.62 (0.46, 5.71)	0.450	1.63 (0.45, 5.94)	0.452

Length								
Absolute LAZ	0.02 (−0.81, 0.86)	0.954	−0.00 (−0.83, 0.83)	0.992	−0.15 (−1.17, 0.88)	0.775	−0.35 (−1.40, 0.71)	0.514
LAZ < −2 SD	0.99 (0.49, 1.99)	0.977	1.03 (0.51, 2.06)	0.941	1.64 (0.70, 3.88)	0.255	1.89 (0.80, 4.46)	0.147

Head circumference								
Absolute HCZ	−0.04 (−0.77, 0.69)	0.911	−0.02 (−0.76, 0.71)	0.948	−0.06 (−1.07, 0.95)	0.905	−0.07 (−1.04, 0.90)	0.888
HCZ < −2 SD	2.08 (0.27, 15.9)	0.480	2.07 (0.27, 16.06)	0.483	1.33 (0.33, 5.38)	0.686	1.33 (0.33, 5.29)	0.684

SD = Standard deviation.

^1^Maternal TDF use defined as any reported TDF-containing ARV regimen used at any time during pregnancy for any amount of time among mothers that used combination ART for HIV treatment or PMTCT while pregnant.

^2^Logistic regression models for binary outcomes and linear regression for continuous outcomes.

^3^Adjusted for maternal age, maternal education level, breastfeeding, gestational age at birth, time since maternal HIV diagnosis, maternal WHO clinical stage, timing of ART initiation (before or during pregnancy), trimester of first combo ART regimen use during pregnancy, and PI-containing ART regimen.
